# Pelvic floor status in opera singers. a pilot study using transperineal ultrasound

**DOI:** 10.1186/s12905-024-02895-6

**Published:** 2024-01-24

**Authors:** Ingrid Volløyhaug, Tuva Semmingsen, Anne-Maria Laukkanen, Clara Karoliussen, Kåre Bjørkøy

**Affiliations:** 1 Department of Obstetrics and Gynaecology, St Olavs Hospital, Trondheim University Hospital, 3250, Torgarden, Trondheim, NO 7006 Norway; 2 Department of Clinical and Molecular Medicine, Norwegian University of Science and Technology, Trondheim, Norway; 3 Griegakademiet, UiB, Bergen, Norway; 4 RDAM, Copenhagen, Denmark; 5 Speech and Voice Research Laboratory, Tampere University, Trondheim, Finland; 6 Trondheim Fysikalske institutt, Trondheim, Norway; 7 Department of Music, NTNU, Trondheim, Norway

**Keywords:** Breath support, Opera singing, Pelvic floor muscle, Pelvic floor muscle contraction, Transperineal ultrasound

## Abstract

**Background:**

Control of pelvic floor muscles (PFM) is emphasized as important to obtain functional breath support in opera singing, but there is not much research that proves PFM function as part of breath support in classical singing. Transperineal ultrasound is a reliable method for quantification of PFM contraction in urogynecology. Our aim was to establish if transperineal ultrasound can be used for observation of movement of the PFM during singing and to quantify pelvic floor contraction.

**Methods:**

Cross sectional study of 10 professional opera singers examined with transperineal ultrasound in the supine position at rest and contraction, and standing at rest and during singing. Levator hiatal area was measured in a 3D rendered volume. Levator hiatal anteroposterior (AP) diameter and bladder neck distance from symphysis were measured in 2D images.

**Results:**

The AP diameter was shortened from supine rest to contraction (15 mm), standing (6 mm) and singing (9 mm), all *p* < 0.01. The bladder neck had a non-significant descent of 3 mm during singing. The mean proportional change in AP diameter from rest to contraction was 24.2% (moderate to strong contraction) and from rest to singing was 15% (weak to moderate contraction).

**Conclusions:**

Transperineal ultrasound can be used to examine the PFM during singing. The classically trained singers had good voluntary PFM contraction and moderate contraction during singing. AP diameter was significantly shortened from supine to upright position, with further shortening during singing, confirming that female opera singers contracted their pelvic floor during singing.

## Brief summary

Transperineal ultrasound can be used to assess pelvic floor muscle contraction during singing. Clasically trained singers contract their pelvic floor muscles during singing.

## Background

Control of pelvic floor muscles (PFM) is emphasized as important to obtain a reliable and functional breath support in opera singing, and some voice pedagogy literature defines the PFM as expiratory musculature working in synergy with the anterolateral abdominal muscles [1]. The PFM is considered to have a dual role providing stability and expiratory force in breathing in synergy with the abdominal muscle in classically trained singers [2]. Still, there is not much research that proves PFM function as part of breath support in classical singing or the role of PFM in improvement of vocal performances, and previous studies are mainly focusing on the activity patterns of neck and abdominal muscles during singing [3–6]. A review from 2020 states that further research is needed to specify the role of the PFM in singing and to ascertain the behavior of the PFM during phonation [2].

Some studies report on PFM activity during breathing, forced expiration and generation of intra-abdominal pressure, situations similar to singing [7, 8]. Digital palpation, perineometry, surface electromyography (sEMG) and magnetic resonance imaging (MRI) have been used to evaluate muscle activity in studies on breathing and forced expiration. Palpation depends on the level of training and personal interpretation of the examiner, perineometry and sEMG could get biased by activation of other muscle groups. MRI is expensive, and it would be difficult to perform in the standing position which is required to study muscle activity during classical singing. One study used transabdominal ultrasound imaging of changes in the shape of the urinary bladder as a proxy for pelvic floor movement during singing [9], but, to our knowledge, no direct visualization of PFM movement during singing has been evaluated.

In urogynecology, transperineal ultrasound imaging of the PFM is validated to assess anatomy and function [10], and ultrasound is a reliable method for quantification of PFM contraction [11]. Ultrasound is available in most gynecological departments, but until now transperineal ultrasound has not been used to assess muscle function in studies focusing on singing and PFM function.

Our aim was to conduct a pilot study to establish if transperineal ultrasound can be used for direct observation of movement of the PFM and urinary bladder during singing and to quantify pelvic floor contraction and bladder movement.

## Methods

### Participants

This was a cross-sectional study of ten professional female classically trained singers examined in January and February 2022 at St. Olav’s Hospital, Trondheim, Norway. The singers were randomly recruited among classically trained professional singers in Middle Norway. Potential participants were contacted by one of the authors (KB) by phone and informed about the study. Inclusion criteria were ongoing careers as opera and concert singers or voice teachers at high school and university level. We aimed for a random sample of parous and nulliparous women. Exclusion criteria were voice health problems, not willing to participate and singers under 25 or over 55 years, as this age range generally is considered the ideal for professional singers.

### Ethical considerations

Study participants gave their informed consent. The project was evaluated by the Regional Ethics Committee, REK vest 396498, and did not need formal approval since it was not regarded as medical research (research regarding health and disease) but a study of physiological functions during singing. It therefore, according to the Regional Ethics Committee, did not apply to the “Act on medical and health research” in Norway.

### Data collection

First, study participants were examined in the supine position standardized for PFM ultrasound with hips and knees semi-flexed. Transperineal ultrasound examination was performed using a Voluson S10 or E8 ultrasound machine (GE Healthcare, Zipf, Austria) with a RAB 4–8-MHz curved array transducer at 85° acquisition angle placed in the midsagittal plane on the perineum. Ultrasound volumes were recorded at rest, PFM contraction and Valsalva maneouvre with forced expiration against a closed glottis. Then the woman was examined in the upright standing position with the legs slightly apart to allow for the ultrasound probe on the perineum. Ultrasound volumes were recorded while the participant was singing short vocalises by changing between two vowels and changing voice intensity within a comfortable range, on four set pitches within a voice range of an octave plus quart in the singer`s middle and higher register. The singers were instructed to use their voice as normal during singing, and they received no information on any contraction or relaxation of the pelvic floor during singing. The examiner (IV) had more than 10 years of experience in pelvic floor ultrasound imaging.

**Fig. 1:**
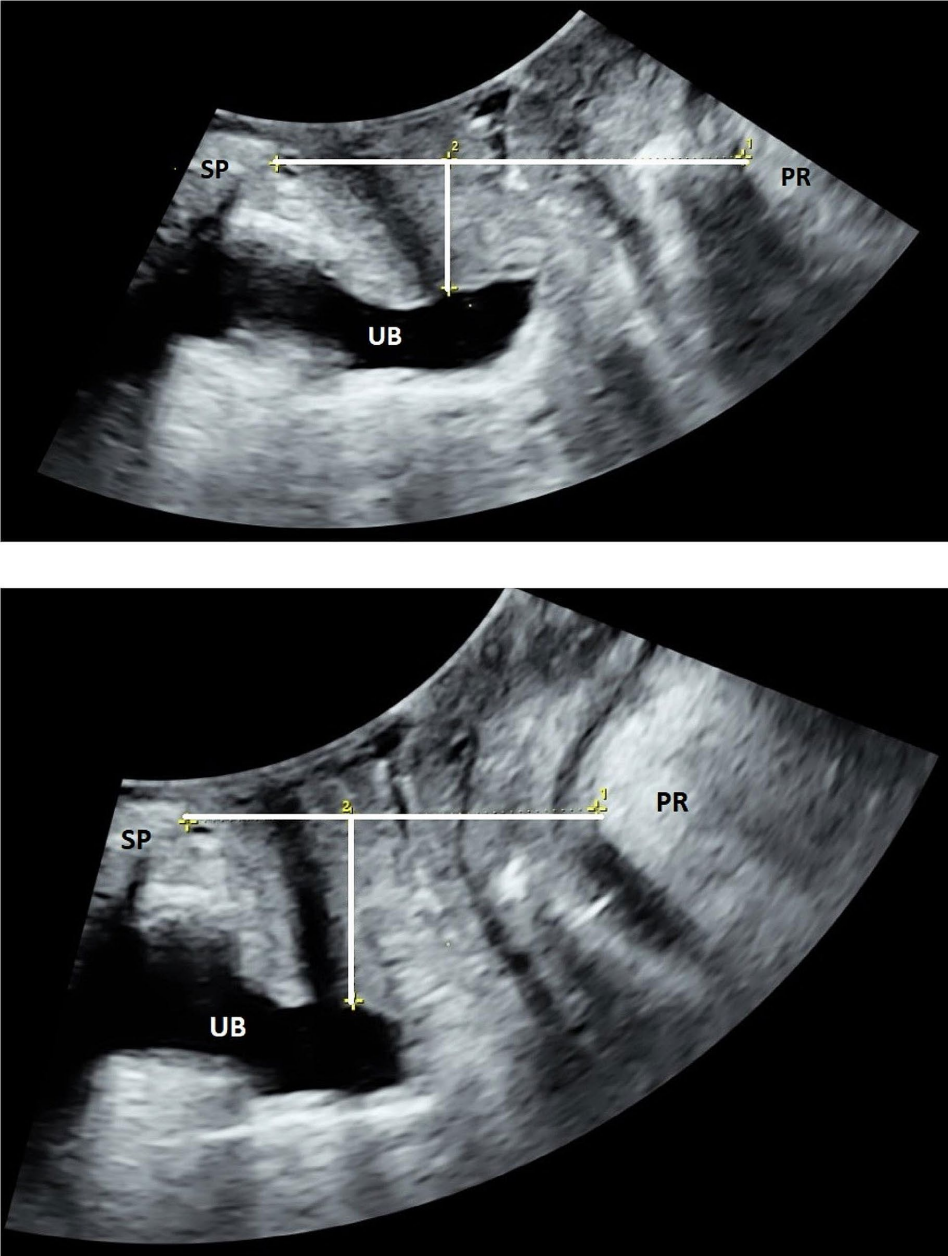
Measurement of the levator hiatal anteroposterior diameter in the plane of minimal hiatal dimensions, from the distal echo of the symphysis pubis (SP) to the puborectalis (PR) bulk behind the rectum (horizontal line) and bladder neck vertical distance from the symphysis (vertical line). Urinary bladder (UB). a) Supine position at rest b) Supine position at contraction

**Fig. 2:**
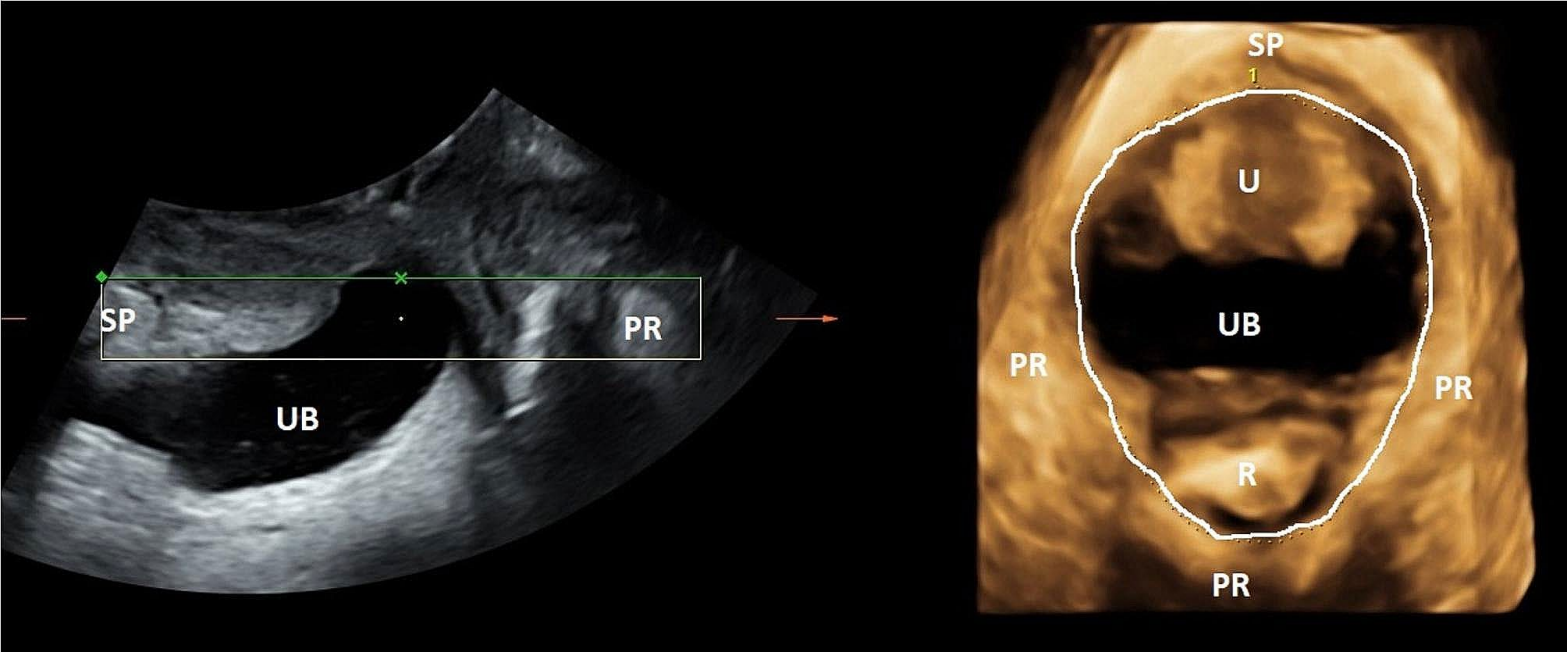
Measurement of the levator hiatal area (thin dotted line) in the rendered 3D image to the right. Symphysis pubis (SP), puborectalis (PR), urethra (U), urinary bladder (UB) and rectum (R)

Offline analysis of the ultrasound volumes was performed by one of the authors (IV) six months later using 4D View version 14 (GE Healthcare). Offline analysis at a later date ensures blinding to whom of the singers each volume belonged to, and interpretation of ultrasound volumes is therefore not biased by clinical data. We used the plane of minimal hiatal dimensions (shortest distance from the distal echo of the symphysis pubis to the anterior edge of the puborectalis muscle behind the rectum) as reference and measured the levator hiatal area, antero-posterior levator hiatal diameter and bladder neck’s vertical distance from the symphysis. All measurements were performed in the supine position at rest, maximum contraction and Valsalva, then standing position at rest and the song rehearsal that resulted in most organ displacement and changes of the PFM [12, 13], see Figs. 1 and 2. We used a previously validated ultrasound contraction scale where proportional change from rest to contraction in 2D levator hiatal anteroposterior diameter classified contraction into: Absent < 1% change, weak 2–14% change, normal 15–29% change and strong > 30% change [11]. Levator avulsion was diagnosed when there was an abnormal muscle attachment to the pubic bone in the three central planes (plane of minimal hiatal dimensions and the two planes 2.5 and 5 mm cranial to this) on tomographic ultrasound imaging at one or both sides [14].

### Statistical analyses

This was a pilot study, and no power calculations were performed. The IBM® SPSS® statistics, version 28.0.0.0. (190) was used in all statistical analyses. Data were tested for normal distribution using Q-Q-plots. We used paired samples t-test to compare ultrasound measurements of the pelvic floor from rest to contraction, upright standing and during singing. The unpaired t-test was used to test for differences between parous and nulliparous women. Pearson’s correlation was used to test for correlation between body mass index (BMI) and PFM contraction (proportional change in levator hiatal anteroposterior diameter) in the supine position and during singing, and we applied the following cut-offs: *r*_*s*_ = 0, no correlation; *r*_*s*_ > 0.3, weak correlation; *r*_*s*_ > 0.5, moderate correlation; *r*_*s*_ > 0.7, strong correlation; *r*_*s*_ = 1, perfect correlation A two-sided p-value < 0.05 was considered statistically significant.

## Results

In total ten professional female classically trained singers, six sopranos and four mezzo-sopranos, participated. Mean age was 35 years, range 25–49, mean parity 1.2, range 0–3, and four were nulliparous. Mean BMI was 24.2 kg/m^2^, range 19–34. Levator hiatal areas, diameters and bladder neck position at rest, during contraction, standing (without voicing) and singing (while standing) are presented in Table 1. Two of the parous women had a unilateral levator avulsion. In one of the singers, the levator hiatal area and AP diameter were not possible to evaluate in the standing position due to motion artifacts. Changes in hiatal area and AP diameter and in the bladder neck’s vertical distance from the symphysis are presented in Table 1. The AP diameter was significantly shortened from supine rest to contraction, standing and singing, and the levator hiatal area decreased significantly from rest to contraction. The bladder neck had a non-significant descent during singing.

**Table 1:** Mean (range) levator hiatal area, levator hiatal anteroposterior diameter and bladder neck (BN) craniocaudal distance from the symphysis in the supine position and standing

	Supine			Standing		Change			
Mean (range)	Rest	Contraction	Valsalva	Rest	Singing	Supine rest to contraction	Supine rest to standing rest	Supine rest to standing singing	Standing rest to singing
Levator hiatal area cm^2^	18.2 12.3–23.5	12.0 8.6–19.3	23.5 12.0-45.3	17.4 8.5–27.3	17.1 9.6–27.0	6.3 *p* < 0.001	1.0 *p* = 0.42	1.3 *p* = 0.29	0.3 *p* = 0.45
Levator hiatal anteroposterior diameter, cm	6.2 5.4–7.5	4.7 4.2–5.7	6.5 5.3-9.0	5.6 4.7–6.6	5.3 4.3–6.5	1.5 *p* < 0.001	0.6 *p* = 0.006	0.9 *p* < 0.001	0.3 *p* = 0.02
BN distance from symphysis, cm	2.6 2.0-3.5	3.1 2.0-4.3	1.3 -1.2–2.8	1.9 -0.7–3.2	1.6 -1.7- 3.0	-0.5 *p* = 0.002	0.7 *p* = 0.109	1.0 *p* = 0.07	0.3 *p* = 0.14

The mean proportional change in levator hiatal AP diameter from rest to contraction was 24.2%, range 17–39%, indicating normal to strong voluntary contraction in all singers.

The mean proportional change in AP diameter from rest to singing was 15%, range 4.6–28.3%, indicating a weak to moderate PFM contraction during singing.

We observed a non-significant weaker contraction in parous women compared to nullipara in the supine position (23.6% vs. 25.0% shortening in AP diameter, *p* = 0.76) and during singing (13.3% vs. 18.5%, *p* = 0.37). We found a moderate positive correlation between BMI and proportional change in levator hiatal AP diameter from rest to contraction *r*_*s*_ =0.65, *p* = 0.041, but this was not significant during singing *r*_*s*_ 0.59, *p* = 0.096.

## Discussion

In this study, we showed that 2D and 3D/4D transperineal ultrasound can be used to examine the PFM during singing. We found that female classically trained singers had good voluntary contraction of the PFM and that they contracted their pelvic floor during singing.

There are few studies with an interdisciplinary view integrating physical medicine with focus on the pelvic floor and singing voice research [2]. Rudavsky et al. used bladder shape distortion visualised with transabdominal ultrasound as a proxy for pelvic floor contraction in ten men and women and found changes in bladder shape during singing, interpreted as a sign of pelvic floor activity [9]. Bedekar et al. conducted a pilot study and showed that singing can be used to train the PFM [15]. They found that activation of the PFMs during singing improved PFM strength assessed by palpation and sEMG after 3–4 weeks of singing practice. Talasz et al. found a positive correlation between PFM function assessed by palpation and expiratory flow in nulliparous women [16]. In another study they found a parallel movement of the thoracic diaphragm and PFM during breathing and coughing, using dynamic MRI, supporting that the PFM plays a role in breathing [17]. In that study they only assessed the craniocaudal displacement of the pelvic floor, and their results could imply that the PFM was passively yielding to the movement of the thoracic diaphragm and increased intraabdominal pressure. Different techniques for assessment of pelvic floor contraction makes direct comparison to other studies challenging, but most studies support an active role of the PFM during breathing, expiration and singing.

We used three different measurements to assess PFM contraction, where levator hiatal anteroposterior diameter measured in the 2D image is the easiest to perform. This technique requires no post-processing of ultrasound volumes, and it has been correlated to palpation and perineometry in previous studies and has shown high interrater interclass correlation (ICC) [11, 18]. Similarly, levator hiatal areas decreased significantly during active contraction. A significant change in 2D levator hiatal AP diameter from standing rest to singing suggests a moderate to strong involvement of the PFM in the singing process. Levator hiatal area also decreased slightly from the supine position to standing, with further decrease during singing, but these changes did not reach statistical significance. We also included a measurement of the bladder neck’s cranio-caudal distance from the symphysis and found that the bladder neck was significantly elevated during voluntary contraction in the supine position. Vertical movement of the bladder neck has been suggested as an indirect measurement of pelvic floor contraction in other studies [13, 19]. In the present study, the bladder neck descended when standing, with a further descent during singing. These latter changes did not reach statistical significance, but we interpret this “paradoxical” descent as an effect of the power of gravity on the urinary bladder and intraabdominal organs when standing up rather than relaxation of the pelvic floor. The bladder neck descended further during singing, probably because of increased intraabdominal pressure created during singing. The PFM contraction observed on singing may be a reflexive response to increased pressure in the abdominal cavity during diaphragm lowering, or muscle contraction might reflect a certain studied voice technique. We speculate that PFM contraction may have prevented a more significant bladder descent during singing.

Another interesting finding was the moderate positive correlation between BMI and PFM contraction. Previous studies have shown that women with higher BMI have higher intraabdominal pressure and increased risk of pelvic floor disorders, especially urinary incontinence [20]. Women with higher BMI might compensate increased intaraabdominal pressure by increasing their maximum PFM contraction. Thre is, however, a lack of studies regarding the possible correlation between BMI and PFM contraction, and further studies are needed.

The main limitation of this study is the small sample size. Multiple regression analysis including confounders such as age, BMI, pelvic floor muscle injury, parity and mode of delivery (caesarean or normal vaginal delivery) was therefore not possible. Previous studies have shown that vaginally parous women with levator avulsions have weaker PFM contraction [21]. This was also observed in the present study, but did not reach statistical significance due to the small sample size. Furthermore, we had no information on PFM training or symptoms of pelvic floor disorders, and we did not ask if the participants contracted voluntarily during singing. We did not inquire about awareness of changes in sensations in the lower waist and pelvic floor region or whether participants had adopted specific techniques or strategies to enhance their singing control in relation to these sensations. The small sample size also limited the possibility to study any correlation between voluntary contraction in the supine position and contraction during singing. We could not correlate grade of contraction with voice intensity or expiratory flow in our study. Only one person examined the ultrasound volumes, and it could be a weakness that the analyses not were repeated by more persons. On the other hand, she had previously been validated against other ultrasound experts and she had over 10 years of experience in pelvic floor imaging.

To our knowledge, this is the first study using direct ultrasound imaging of the PFM on opera singers both in the supine position and during singing in a natural, standing position. Other strengths are that the singers were examined in both the supine and standing position and we used validated measurements for assessment of PFM contraction. Ultrasound minimises the bias of other techniques, such as the examiners subjective interpretation by palpation of the PFM and coactivation of other muscles by perineometry and sEMG. The inclusion of both parous and nulliparous women increases the external validity of the results.

We have confirmed that ultrasound is a feasible tool in studies of the PFM during singing. The examiner experienced that transperineal ultrasound was more difficult to perform in the standing position during singing, as the women moved slightly during singing and the examiner had to follow the singer’s movement. Nonetheless, most of the ultrasound volumes were of good quality, and we advocate the use of transperineal ultrasound in future studies of PFM action during singing.

This study forms the basis for larger scale studies among singers, and it has implications also for other professions in whom breath control and pelvic floor support is thought to be important, such as wood wind and brass musicians. Pelvic floor ultrasound could be used to test if musically untrained persons contract the pelvic floor similarly during singing and if contraction is correlated with voice quality. One previous study found different contraction patterns in abdominal muscles in untrained versus trained singers [4], and another study found different function of the abdominal muscles in singers with and without functional voice disorders [6]. Future studies should include questions about pelvic floor disorders and symptom scores in classically trained singers. Previous studies have shown that female athletes report high prevalence of pelvic floor disorders and that they use different strategies to control the symptoms [22–24]. We could imagine that opera singers are similar to athletes since controlled straining is part of the singing technique, but on the other hand, opera singers could have less PFD because of focus on the PFM during education and good PFM contraction.

## Conclusions

In conclusion, we confirmed that 2D and 3D/4D transperineal ultrasound can be used to examine the pelvic floor during singing. The classically trained singers had good voluntary pelvic floor muscle contraction and moderate contraction during singing. Anteroposterior levator hiatal diameter was significantly shortened from supine to upright position, with further shortening during singing, reflecting contraction. This confirms that female opera singers are contracting their pelvic floor as breath support during singing.

## Data Availability

The datasets analysed during the current study are available from the corresponding author on reasonable request.

## Abbreviations

AP: Anteroposterior
BMI: Body mass index
ICC: Interclass correlation
MRI: Magnetic resonance imaging
PFD: Pelvic floor disorders
PFM: Pelvic floor muscle
sEMG: Surface electromyography
2D: Two dimensional
3D/4D: Three and four dimensional
